# Inducible and combinatorial gene manipulation in mouse brain

**DOI:** 10.3389/fncel.2015.00142

**Published:** 2015-04-21

**Authors:** Godwin K. Dogbevia, Ricardo Marticorena-Alvarez, Melanie Bausen, Rolf Sprengel, Mazahir T. Hasan

**Affiliations:** ^1^Department of Molecular Neurobiology, Max Planck Institute for Medical ResearchHeidelberg, Germany; ^2^Institute of Experimental and Clinical Pharmacology and Toxicology, University of LübeckLübeck, Germany; ^3^NeuroCure Cluster of Excellence, Charité-UniversitätsmedizinBerlin, Germany

**Keywords:** tetracycline inducible systems, Cre mediated recombination, rAAVs, brain circuits, learning and plasticity, neurological diseases

## Abstract

We have deployed recombinant adeno-associated viruses equipped with tetracycline-controlled genetic switches to manipulate gene expression in mouse brain. Here, we show a combinatorial genetic approach for inducible, cell type-specific gene expression and Cre/loxP mediated gene recombination in different brain regions. Our chemical-genetic approach will help to investigate ‘when’, ‘where’, and ‘how’ gene(s) control neuronal circuit dynamics, and organize, for example, sensory signal processing, learning and memory, and behavior.

## Introduction

Brain is a complex organ, with highly organized genetic and cellular programs that engages neural circuits to generate synaptic specificity (Yogev and Shen, 2014) and plasticity (Citri and Malenka, 2008; Huganir and Nicoll, 2013), controlling a diverse range of biological functions, for example, sensory signal detection, movement, decision-making, and learning and memory (Tonegawa et al., 2003; Kandel et al., 2014).

An attractive hypothesis is that local and distributed neural circuits encode biological functions. To test this hypothesis, it is imperative to ‘causally’ link the anatomical and functional organization of the neural circuits to biological functions. Manipulating circuit dynamics (Deisseroth, 2014) is paving the way to understand the operating principles of normal and aberrant brain states. Within this framework, understanding the role of gene function(s) in circuit dynamics and biological functions is an important first step in determining the rules and mechanisms that orchestrate changes across different synaptic connections throughout the brain. The application of inducible gene expression systems to manipulate various genes, the building blocks of circuit function(s), in identified brain regions and cell types will help to reveal the molecular and cellular mechanisms that control and regulate biological functions.

The tetracycline (tet) inducible systems (Sprengel and Hasan, 2007) are adequately suitable for investigating various biological processes in the brain. The tet systems have three key components: (1) The transactivator (tTA; Gossen and Bujard, 1992) or the reverse tTA (rtTA; Urlinger et al., 2000), which are artificially designed potent transcription factors that can be expressed constitutively in cells under control of a ubiquitous or a cell type specific promoter. (2) A tet responsive minimal promoter (either unidirectional P_tet_ or bidirectional P_tet_bi; Baron et al., 1995; Sprengel and Hasan, 2007), which become strongly activated upon binding to tTA/rtTA. (3) A chemical inducer, doxycycline (Dox; Bocker et al., 1981), that can rapidly cross the blood–brain-barrier (Bocker et al., 1981), and controls the binding of tTA/rtTA to P_tet_/P_tet_bi. The tTA and rtTA, commonly called Tet-off and Tet-on, respectively, are complementary systems (Sprengel and Hasan, 2007). Without Dox, tTA binds to P_tet_/P_tet_bi to activate gene expression. However, when Dox binds to tTA, a conformational change in tTA disables its ability to bind P_tet_/P_tet_bi, inactivating gene expression. The reverse is the case for rtTA; only the Dox-rtTA complex can bind to P_tet_/P_tet_bi to activate gene expression, which becomes inactivated when Dox unbinds rtTA.

In particular, the tTA system has been used in transgenic rodents to repetitively turn ‘on’ and ‘off’ gene expression by Dox withdrawal and addition, respectively, (Hasan et al., 2001). The tet systems also allow for gene expression-amplification (Hasan et al., 2004). For inducible gene fragment deletion by Cre/loxP mediated recombination, the tTA system is, however, quite cumbersome. In transgenic tTA mouse models, for Cre/loxP mediated gene manipulation, Cre gene expression in unborn transgenic animals is switched-off by treating pregnant females with Dox. In addition, before investigating specific biological processes, gene expression in newborn pups, is also switched-off by Dox. During both prenatal and pup rearing stages, the Dox reaches the unborn and newly born animals via placenta and milk, respectively. There are two problems with such an approach: (1) Dox availability to the brain via milk is not efficient, which increases the likelihood for undesired Cre/loxP mediated recombination and (2) Dox provided via the placenta in embryos, before blood–brain-barrier is formed, strongly suppresses Cre gene expression, which tend to silence P_tet_/P_tet_bi, disabling gene activation by Dox withdrawal at later stages (Zhu et al., 2007). With excessive accumulation of Dox in tissues during prenatal development, and slow time course of Dox clearance, it can take several months before Cre gene expression can be switched-on by Dox removal. Even after Dox clearance, Cre gene expression in a majority of neurons remains switched-off (Zhu et al., 2007). However, the rtTA system provides a potential solution to this setback. With the rtTA system, Dox activates gene expression via P_tet_/P_tet_bi (Zhu et al., 2007). Although the rtTA system works well in various body tissues, its performance is inefficient in the brain of transgenic mammals (Zhu et al., 2007).

In our previous study, we systematically investigated this problem and showed that when tet promoters (P_tet_/P_tet_bi) are switched-off during development, they becomes epigenetically silenced in neurons (Zhu et al., 2007). However, introducing a P_tet_bi module in recombinant adeno-associated viruses (rAAVs), which remain largely episomal (Schnepp et al., 2005) in cells, epigenetic silencing of P_tet_bi in neurons can be avoided (Zhu et al., 2007). Taking advantage of this discovery, we developed AAVs for reliable and efficient inducible gene expression in brain (Zhu et al., 2007). Our approach is based on two different viruses, which can be delivered in the brain by stereotactic injection (Zhu et al., 2007; Wallace et al., 2008). The first virus delivers in neurons the rtTA (rtTA2-nM2; Zhu et al., 2007) gene under a pan-neuronal human synapsin promoter (Zhu et al., 2007; Cambridge et al., 2009; Hasan et al., 2013). The second virus delivers P_tet_bi to simultaneously express two different genes (tdTomato and iCre; Cambridge et al., 2009; Hasan et al., 2013) in a Dox-controlled, rtTA dependent manner. The AAVs offer other key advantages as well; they allow for precise and combinatorial targeting of brain region(s) at different stage over the course of biological studies, something that is currently not possible with traditional transgenic animal models. The availability of different AAV serotypes and synthetic capsid proteins is expanding the versatility of the AAV-based approach by targeting diverse cell types (Wang et al., 2011; Drouin and Agbandje-McKenna, 2013), with limited or no immune response (Drouin and Agbandje-McKenna, 2013). Therefore, AAVs have great potentials for investigating basic brain functions and are ideally suited as gene therapy vectors to treat neurological diseases (Bourdenx et al., 2014).

In this study, we deployed AAVs for Dox-controlled, rtTA-dependent expression of two genes, Cre (Shimshek et al., 2002) and tdTomato (tdTOM; Shaner et al., 2005). The Cre recombinase allows for Cre/loxP mediated gene manipulation (Capecchi, 2005) of different brain regions and tdTOM marks the cells for live imaging. Our experimental approach will facilitate investigation of gene function(s) in diverse biological processes, including learning and memory (Hasan et al., 2013).

## Materials and Methods

### Mice

C57BL/6N (Charles River, Sulzfeld, Germany) and gene-targeted *Gt(ROSA)*^26Sortm1Sor/J^ mice were housed under standard conditions in a 12 h light/dark cycle in Makrolon cage type-2A with food and water. All animal procedures were performed with permission and in accordance with German governmental regulation on animal experimentation (Regional Council Karlsruhe, Germany: 35-9185.81/G171/10).

### Plasmid Constructs

The plasmids pAAV-hSYN-rtTA2-nM2 (Zhu et al., 2007; Hasan et al., 2013) and pAAV-P_tet_bi-iCre/tdTOM (Cambridge et al., 2009; Hasan et al., 2013) used here have been described previously. The GenBank Accession Numbers for these plasmids are KP893810 and KP893811, respectively.

### Virus Purification

Serotypes 1 and 2 rAAVs were generated by transfection into HEK293 cells and purified as described previously (Zhu et al., 2007). In brief, each AAV plasmid, pAAV-hSYN-rtTA2-nM2 (Zhu et al., 2007; Hasan et al., 2013) and pAAV-P_tet_bi-iCre/tdTOM (Cambridge et al., 2009; Hasan et al., 2013) was individually co-transfected with pDp1 (for serotype 1) and pDp2 (for serotype 2) helper plasmids at a ratio of 2:1:1 per 15-cm plate (25 mg pAAV, 12.5 mg pDp1 and 12.5 mg pDp2) into HEK293 cells by the DNA/Ca^2+^PO_4_ mediated co-precipitation (Chen and Okayama, 1987). We plated HEK cells on twenty 15-cm plates with 25 ml of DMEM medium per plate. On the day of transfection, the cells should be 50% confluent. Forty-eight hours after transfection (50% efficiency), cells were harvested and collected in 50 ml tubes and pelleted by centrifugation at 2000 rpm for 5 min. The cell pellets were suspended in 45 ml of suspension buffer (150 mM NaCl, 20 mM Tris.HCl, pH 8.0) and 0.5 ml of 10% sodium deoxycholate (NaDOC) was added (final concentration of 0.5% NaDOC). To the well-mixed cell lysate was added benzonase (40 U/ml), mixed well, and incubated at 37C^∘^ for 60 min. At this stage, cell lysates were frozen at -70^∘^C. Before purification, cell lysates were thawed at room temperature and centrifuged at 4000 rpm for 15 min, supernatant collected in a new 50-ml tube and frozen again at -70^∘^C overnight. The next day, supernatant was thawed and centrifuged at 4000 rpm for 15 min. A clear supernatant was passed through a pre-equilibrated 1-ml heparin column (Amersham, Freiburg, Germany) using a pump device. The column was serially washed with 20 ml of 100 mM NaCl, 20 mM Tris.HCl (pH 8), 1 ml of 200 mM NaCl, 20 mM Tris.HCl (pH 8), and 1 ml of 300 mM NaCl, 20 mM Tris.HCl (pH 8). The virus was eluted with 1.5 ml of 400 mM NaCl, 20 mM Tris.HCl (pH 8), 3 ml of 450 mM NaCl, 20 mM Tris.HCl (pH 8), and 1.5ml of 500 mM NaCl, 20 mM Tris.HCl (pH 8). The eluted virus was pooled into a 15-ml Amicon Ultra concentrator (Millipore) and filled to the top with 1x PBS. After centrifugation at 2000 rpm for 10 min, the flow through was discarded, and the reservoir was re-filled with 1x PBS. This procedure was repeated a total of three times. The concentrated viruses (200 μl) were sterilized through a 0.2 μm small size filter device, aliquoted into eppendorf tubes, and stored at -80^∘^C until use. A small sample of purified virus (10 ml) was analyzed by SDS-PAGE. The gel was stained for 45 min with Coomassie blue and destained for another 45 min, and washed 5x with water, until protein bands were clearly visible. With successful virus purification, three bands corresponding to the viral capsid proteins can be seen, with expected molecular weights of 87 kDa (VP1), 73 kDa (VP2), and 62 kDa (VP3).

### Responder Virus with Minimal Leakiness

To reduce leakiness of the P_tet_bi responder virus (rAAV-P_tet_bi-iCre/tdTOM; Cambridge et al., 2009; Hasan et al., 2013), highly concentrated virus were titered [genomic titer of 1–3 × 10^13^ vector genome (vg) per milliliter]. The responder virus (rAAV-P_tet_bi-iCre/tdTOM) was diluted 1:1, 3:1, 10:1, and 30:1 with respect to the activator virus (rAAV-hSYM-rtTA2-nM2; Zhu et al., 2007; Hasan et al., 2013). About 500 nl of virus cocktail, rAAV P_tet_bi-iCre/tdTOM, and rAAV-hSYN-rtTA2-nM2, was added directly and gently on top of hippocampus organotypic brain slices. Medium was changed after 5 days and, subsequently, every 3 days. Two weeks after virus infection, two slices were incubated with 1 μg/ml Dox in the medium for 48 h and two slices were kept without Dox (control). Forty-eight hours after Dox addition, tissues were washed in warm 1x PBS and fixed in 4% PFA. Fixed tissues were imaged for tdTOM expression. The diluted virus cocktail that showed undetectable tdTOM expression (without Dox) and strong tdTOM expression with Dox was chosen for *in vivo* application.

### Stereotactic Virus Injection in Mouse Brain

C57BL/6N and *Gt(ROSA)*^26Sortm1Sor/J^ mice were deeply anesthetized with ketamine (100 mg/kg) and xylazine (5 mg/kg), and were secured in a Kopf stereotaxic setup (Kopf Instruments, Tujunga, CA, USA). The foreskin on the skull was cut open to expose the skull. A small hole (50–100 μm) was made through the skull using a dental drill. A glass pipette delivered approximately 200 nl of virus cocktail by injection into different brain regions; cortex and hippocampus. The coordinates used for the injections are with reference to the bregma: cortex (–1.70 mm bregma, 1.5 mm lateral, 500 μm deep) and hippocampus (–1.70 mm bregma, 1.5 mm lateral, 1.5 mm deep). After virus injection, the skin was sutured and the wound was disinfected. Virus injected mice were kept on a heating blanket at 37^∘^C until they woke up, and were fed wet food during recovery.

### Dox Treatment *In Vivo*

For intraperitoneal injection, stock Dox solution (5 mg/ml in 0.9% NaCl) was prepared. Effective dose is 50 μg of Dox per gram body weight (10 μl of Dox per 1 g of animal weight).

### Fixed Brain Slices

Mice were anesthetized by isoflurane inhalation followed by intracardial perfusion with PBS and 4% paraformaldehyde (PFA). Brains were removed and post-fixed in 4% PFA for 2 h at 4^∘^C, kept in 1x PBS at 4^∘^C overnight, washed 3x with PBS and embedded in 2.5% agarose (in 1x PBS). Typically, we prepared vibratome (VT1000S, Leica, Wetzlar, Germany) brain slices, which were 60–100 μm in thickness. Coronal brain slices were stored in 1x PBS at 4^∘^C.

### Immunohistochemistry

Brain slices were incubated in 4% normal goat serum (supplemented with 1% BSA, 0.3% TritonX-100) for 15 min and then incubated overnight at room temperature with primary Cre-antibody (mouse monoclonal anti-Cre; 1:1000, Covance, Germany) diluted in PBS/ 1% BSA/ 1% normal goat serum/ 0.3% TritonX-100. The next day, brain slices were washed 2x in 1x PBS/ 0.3% BSA/ 0.1% TritonX-100 (D2), followed by incubation with anti-mouse FITC secondary antibody (1:200, Jackson Immuno Research) for 1 h at room temperature. Brain slices were washed in 1x D2 buffer followed by a single wash in 1x PBS and mounted with Aqua Poly/Mount on glass slides with coverslips.

### β-Galactosidase Assay

rAAV-hSYN-rtTA2-nM2 (Zhu et al., 2007; Hasan et al., 2013) and rAAV-P_tet_bi-iCre/tdTOM (Cambridge et al., 2009; Hasan et al., 2013) were co-injected into the hippocampus and cortex of *Gt(ROSA)*^26Sortm1Sor/J^ mice. Two weeks after virus injection, mice were either not treated or treated with Dox by a single intraperitoneal injection. After 48 h, animals were sacrificed, brains were fixed and sliced as described above. To visualize β-galactosidase activity, fixed brain slices were incubated in X-gal solution (5 mM K_4_Fe(CN)_6_, 5 mM K_3_Fe(CN)_6_, 2 mM MgCl_2_, 2 mg/ml X-Gal in dimethylformamide/PBS) at room temperature for 30–60 min. Sections were washed 3x in PBS, 1x in 10 mM Tris-HCl pH 7.5 and, subsequently, mounted on glass slides with Aqua Poly/Mount (Polysciences, Inc., Warrington, PA, USA) and protected with cover slips and later imaged.

### Imaging

Light and fluorescence imaging were performed with Zeiss Axioplan-2 (Carl Zeiss, Jena, Germany) with the camera system AxioCam HRC with magnifications ranging from 2.5× to 40× dry or 63× oil immersion objectives (software: Axiovision 4.8.1) and a compact light source (Leistungselectronic Jena, Germany) with 488 nm and 568 nm filters. Confocal images were acquired with Zeiss LSM PASCAL confocal laser-scanning microscope equipped with an Argon laser (457, 476, 488, and 514 nm) and a Helium Neon laser (543 nm) with objectives 5×–40× dry and 63× oil-immersion objective. Images were analyzed with ImageJ and LSM image browser.

## Results

### Gene Activation with Tet Promoters

**FIGURE 1:**
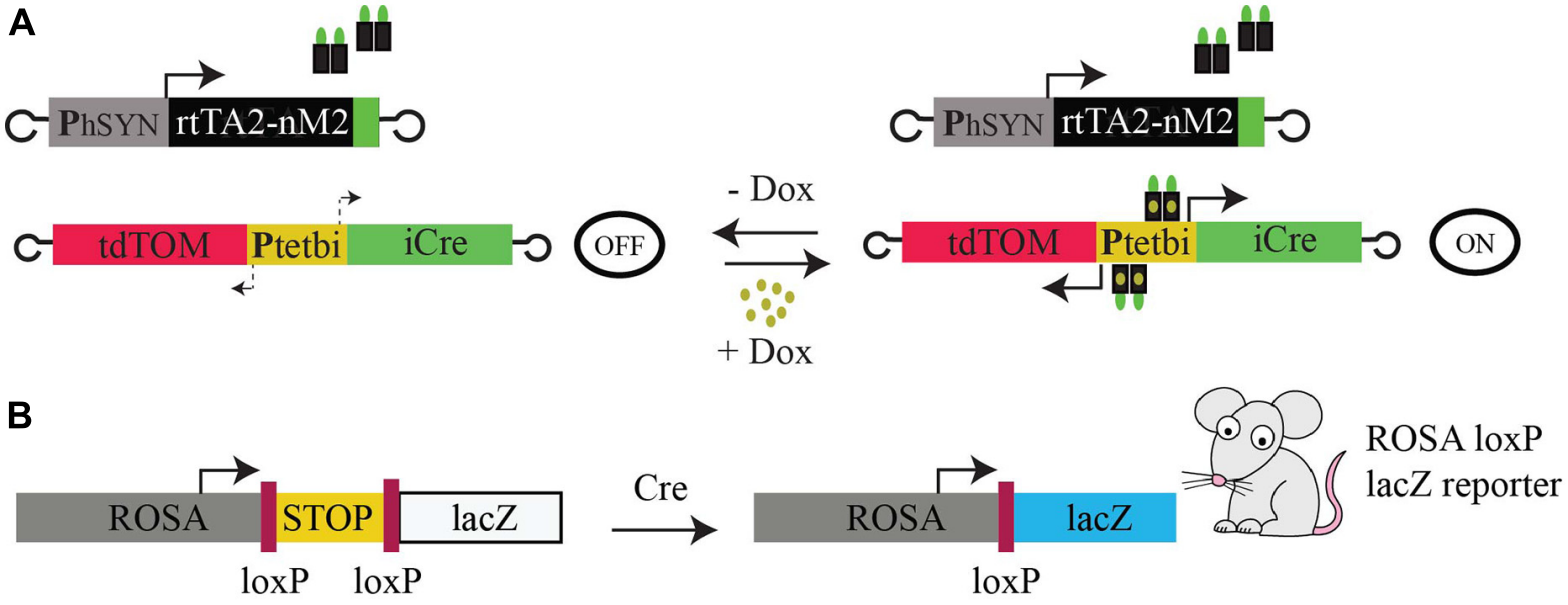
**Operating principles. (A)** Schematics of rAAV-rtTA system; a virus with a human synapsin promoter for constitutive, pan-neuronal specific rtTA expression and another virus with a bidirectional tetracycline (tet) promoter (P_tet_bi) driving two different genes (tdTOM and iCre) in opposite directions. Binding of doxycycline (Dox) to rtTA enables it to bind to P_tet_bi, and gene expression is bidirectionally switched-ON. In the absence of Dox, rtTA is unable to bind P_tet_bi and gene expression is switched-OFF. **(B)** Schematic of *the lacZ* Cre-dependent reporter in *Gt(ROSA)*^26Sortm1Sor/J^ mice. The ROSA promoter drives the expression of the lacZ gene, but this expression is blocked by transcriptional terminator sequences (STOP), which are flanked by loxP sites. The terminator STOP fragment is removed by Cre/loxP mediated gene deletion to activate the expression of the lacZ gene.

**FIGURE 2:**
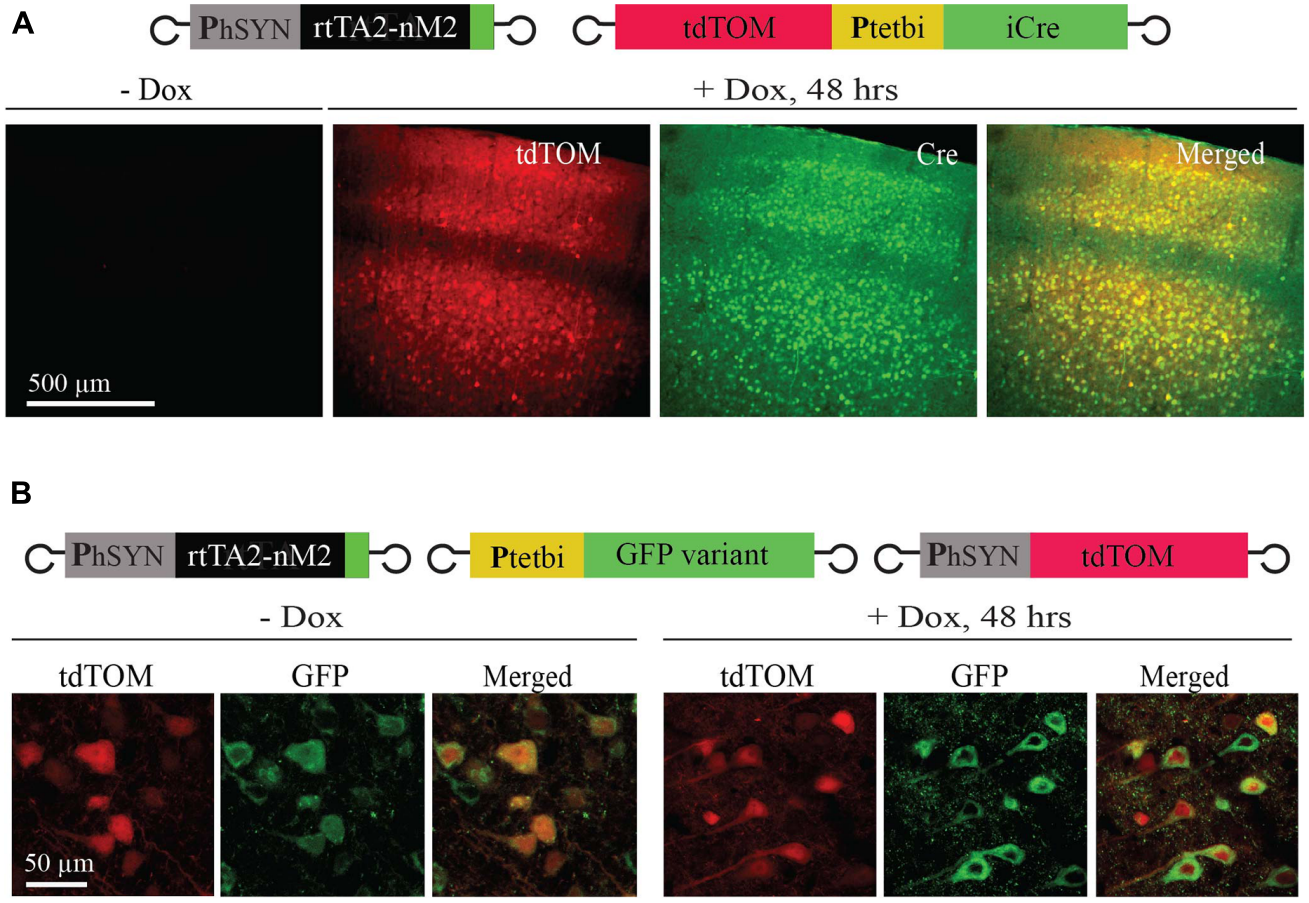
**P_tet_bi assisted inducible gene expression. (A)** After Dox treatment, the P_tet_bi-iCre/tdTOM vector enabled robust tdTOM and iCre co-expression in an rtTA dependent manner (right), but not without Dox (left). Both tdTOM and iCre expression was colocalized in cortical neurons (right; merged image). **(B)** Expression under the control of P_tet_bi-GFP-variant vector alone was similar, both with and without Dox, and it compared well with tdTOM expression under the human synapsin promoter.

To achieve Tet-inducible gene expression and gene deletion in mouse brain, we used two rAAVs, rAAV-hSYN-rtTA2-nM2 (Zhu et al., 2007; Hasan et al., 2013) and rAAV-P_tet_bi-iCre/tdTOM (Zhu et al., 2007; Cambridge et al., 2009; Hasan et al., 2013), and loxP-STOP-loxP-lacZ reporter (*Gt(ROSA)*^26Sortm1Sor/J^; Soriano, 1999) transgenic mice (**Figures 1A,B**). The first virus (rAAV-hSYN-rtTA2-nM2) is equipped with the human synapsin promoter to drive rtTA (rtTA2-nM2) expression. The second virus (rAAV-P_tet_bi-iCre/tdTOM) has a bidirectional tet promoter (P_tet_bi) to express two responder genes, iCre (Shimshek et al., 2002) and tdTomato (Shaner et al., 2005; tdTOM; **Figure 1A**). These two viruses were injected in the cortex of four wild-type mice. Two weeks later, two mice were treated with Dox by a single intraperitoneal injection and the other two mice were not treated with Dox. We found that there was robust expression of tdTOM and Cre recombinase 2 days after Dox treatment. In the cortex, expression was largely restricted to neurons of layer 2/3 and 5, which was evenly distributed in soma and dendrites, but there was little or no expression in cortical layers 4 and 6 neurons. No tdTOM and Cre expression was detectable in mice without Dox. The two transgenes (tdTOM and iCre) under a P_tet_bi also showed faithful co-expression (**Figure 2A**). Interestingly, when one gene (GFP variant) was placed under control of a P_tet_bi (rAAV-P_tet_bi-GFPvariant, called here rAAV-uni-P_tet_-GFPvariant), strong gene expression was observed, even without Dox (**Figure 2B**), and was quite comparable to tdTOM expression under a constitutive human synapsin promoter (**Figure 2B**). We made this observation with three different GFP-linked genes with a similar rAAV-uni-P_tet_-GFPvariants (data not shown). These results suggest that the placement of two genes flanking a P_tet_bi module allow for Dox-controlled, rtTA-dependent regulated gene expression, but not when only one gene is placed under a structurally intact P_tet_bi.

### Time Course of Gene Activation

**FIGURE 3:**
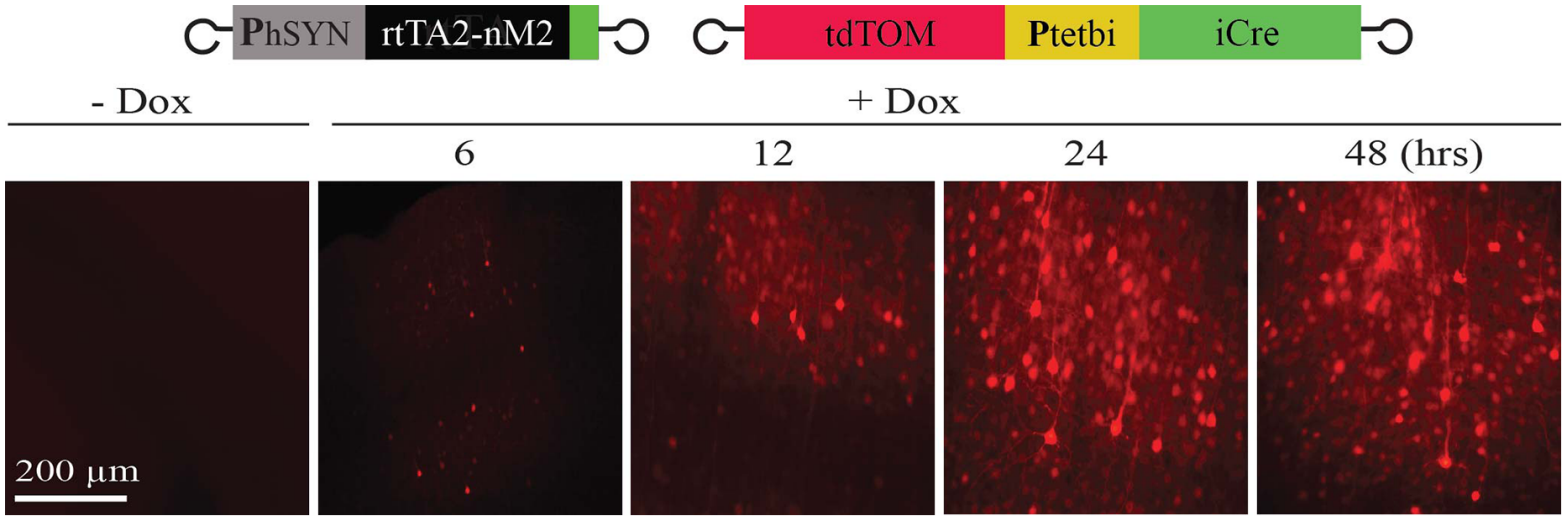
**Time course of gene activation.** After Dox treatment, gene activation was detected *in vivo* as early as 6 h, which reached maximum levels in the cortex after 24 h.

To investigate the time course of Dox-controlled, rtTA-dependent gene activation *in vivo*, the two viruses (Zhu et al., 2007; Cambridge et al., 2009; Hasan et al., 2013; rAAV-hSYN-rtTA2-nM2 and rAAV-P_tet_bi-iCre/tdTOM) were co-injected into the cortex of 10 wild-type mice. Two weeks later, mice were divided into five groups (two mice per group). The first group was without Dox (control) and the other four groups were treated with Dox with a single intraperitoneal injection, and tdTOM expression was analyzed after 6, 12, 24, and 48 h in fixed brain slices. While tdTOM expression was not detectable in mice without Dox (**Figure 3**, left panel), Dox-treated mice showed strong expression as early as 6 h, and maximum expression was reached after 24 h (**Figure 3**).

### Inducible Gene Expression and Cre/*Lox*P Mediated Gene Recombination

**FIGURE 4:**
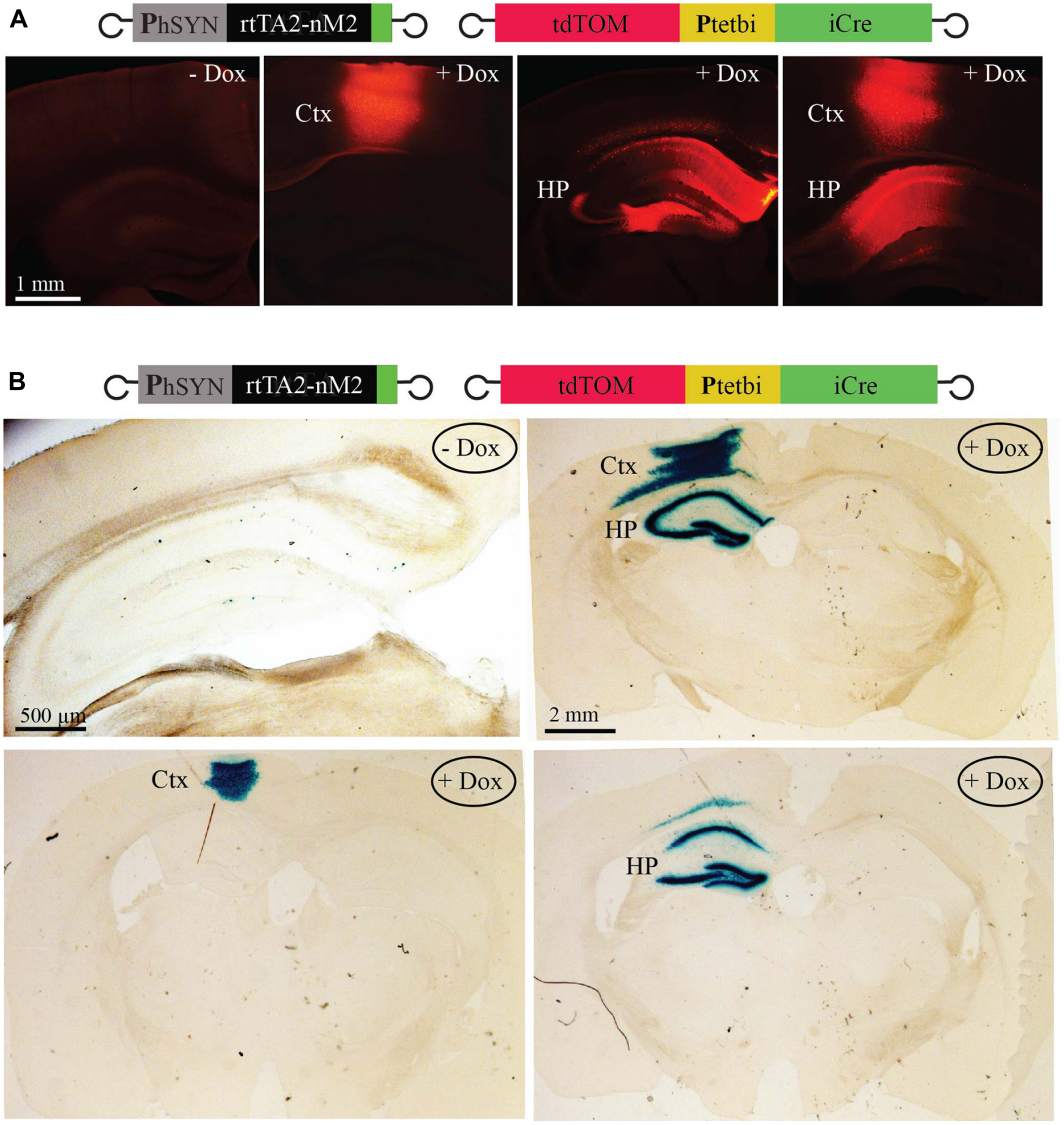
**Inducible, Cre/loxP mediated gene recombination. (A)** With precisely targeted virus injection, induced tdTOM expression in cortex alone, hippocampus alone and both cortex and hippocampus. **(B)** Cre/loxP mediated recombination to activate the lacZ gene in different singly targeted brain region and in combination of two regions. Expression of β-galactosidase was assayed by X-gal staining. Very little recombination was detectable without Dox (left panel). Ctx, cortex; HP, hippocampus.

To achieve neuron- and brain region-specific inducible gene expression and Cre/loxP mediated gene recombination, viruses (Zhu et al., 2007; Cambridge et al., 2009; Hasan et al., 2013; rAAV-hSYN-rtTA2-nM2 and rAAV-P_tet_bi-iCre/tdTOM) were co-injected in two different brain regions in three different combinations; cortex alone, hippocampus alone, and both cortex and hippocampus (two mice per group). Two weeks after virus injection, mice were treated with Dox by a single intraperitoneal injection. Two mice served as controls (without Dox). Two days after Dox injection, fixed brain slices showed strong tdTOM expression (**Figure 4A**). In the hippocampus, expression was widespread in the dentate gyrus granule cells, and the CA1/CA3 pyramidal neurons. No tdTOM expression was detected in mice without Dox (**Figure 4A**). To test for Cre/*lox*P gene recombination in specific brain regions in the *Gt(ROSA)*^26Sortm1Sor/J^ mice, viruses (rAAV-hSYN-rtTA2-nM2 and rAAV-P_tet_bi-iCre/tdTOM) were co-injected again in three different combinations; cortex alone, hippocampus alone, and both cortex and hippocampus (two mice per group). Two weeks after virus injection, mice were injected with a single dose of Dox and two mice were used as control (without Dox). Forty-eight hours later, fixed brain slices showed strong β-galactosidase activity by X-gal staining (**Figure 4B**), indicating that Cre/*lox*P mediated gene recombination occurred efficiently in Dox-treated mice. It should be noted, however, that if the viruses do not reach the entire brain structure, as in the case of hippocampus alone example, a small region would remain untargeted (see CA2 region, **Figure 4B**, lower right panel). Control mice, without Dox, showed recombination in only a few cells (**Figure 4B**, left panel).

## Discussion

Here, we report a versatile genetic approach that takes advantage of two rAAVs for inducible, brain region, and cell type specific gene manipulation. Our proof-of-principle approach is currently based on the tet inducible gene expression system, namely the rtTA system, for Dox-induced Cre recombinase expression to induce Cre/loxP mediated gene recombination. The first virus is equipped with a cell type specific promoter to express rtTA (rtTA2-nM2; Zhu et al., 2007). The second virus harbors a bidirectional tet promoter (P_tet_bi) to co-express two different genes in opposite orientations (Baron et al., 1995). The use of a fluorescent protein in P_tet_bi, for example, tdTOM (Shaner et al., 2005), helps to monitor the expression of a second gene in the targeted brain region(s). Inducible activation of gene expression via a P_tet_bi requires an rtTA and an inducer, Dox, which can be delivered to the animals by an intraperitoneal injection (Zhu et al., 2007; applied here) and/or in the drinking water (not applied here). We found that Dox-induced, rtTA-dependent P_tet_bi mediated gene expression in targeted neurons can be detected within, at least, a few hours. Our AAVs are of a hybrid serotype (1/2), which appear to largely target layer 2/3 and layer 5 neurons, but not layer 4 and layer 6 neurons. We have not observed retrograde labeling with the AAV1/2.

We demonstrate the applicability of virus approach for inducible gene expression and Cre/loxP mediated gene recombination in the brain of lacZ transgenic reporter mice [*Gt(ROSA)*^26Sortm1Sor/J^; Soriano, 1999]. It is well established that the lacZ transgenic reporter mice is a reliable model for natural floxed genes. Our approach can also be extended to overexpress wild-type and mutant genes (Eschbach and Danzer, 2014) and interference RNA (Murphy et al., 2013; Ramachandran et al., 2013). With stereotactic virus injection, we can achieve long-term gene expression in either a single or multiple brain regions, enabling systematic investigation of how different brain regions participate in various biological processes, for example, learning and memory.

We found that rAAVs equipped with minimal tet promoters (P_tet_/P_tet_bi) are not without problems. First, P_tet_/P_tet_bi have very low levels of intrinsic transcriptional activity, which is one source of leakiness. In most cases, this is not a major problem. However, with a high virus titer, rAAV-P_tet_bi-iCre/tdTOM (Zhu et al., 2007; Cambridge et al., 2009; Hasan et al., 2013) alone (without rtTA and without Dox), for example, can produce enough Cre recombinase protein in a small number of neurons, particularly, at the virus injection brain site(s) to allow for Cre/loxP mediated gene recombination (data not shown). It is therefore important to serially dilute P_tet_bi viruses with a constant amount of an rtTA (or tTA) virus and only apply an optimal virus cocktail (P_tet_bi + rtTA) for efficient and reliable Dox-induced, rtTA-depednent Cre/loxP mediated gene recombination.

The other issue is that the two flanking inverted terminal repeats (ITRs; Bohenzky et al., 1988) in rAAVs appear to have a cryptic enhancer activity. We found that when one gene is placed under a P_tet_bi (equivalent to P_tet_), it becomes highly active. We reasoned that this increase in P_tet_bi activity was influenced by a nearby ITR. It is known that minimal promoters including P_tet_/P_tet_bi can trap enhancers (Stanford et al., 2001). We thus speculate that an ITR can act in cis to increase the basal activity of the minimal tet promoter. This phenomenon might also explain why gene expression modules flanked by ITR sequences in transgenic zebrafish enabled stable and uniform gene expression (Hsiao et al., 2001), throughout generations, but not without ITRs (Hsiao et al., 2001). In our approach, two different genes (iCre and tdTOM) in rAAV-P_tet_bi-iCre/tdTOM (Zhu et al., 2007; Cambridge et al., 2009; Hasan et al., 2013) appear to shield P_tet_bi from ITR enhancer-like activity; adding DNA sequences of more than 700 bp in between a P_tet_bi appears to minimize the influence of the two ITRs onto P_tet_bi. Clearly, the proposed role of an ITR as an enhancer should be investigated more systematically. It still remains an open question, however, if different gene fragments might insulate P_tet_ from an adjacent ITR to a different extent.

Ours is not the first example of a two AAV approach for inducible gene expression. A previous study elegantly used tet transsilencer (tTS) and rtTA on one virus and a P_tet_ (unidirectional) on another virus to express GFP in a Dox-controlled, rtTA-dependent manner (McGee Sanftner et al., 2001). In that system, in the absence of Dox, the tTS prevents leaky expression by P_tet_, possibly by blocking the ITR enhancer-like activity. With Dox treatment, tTS come off, and rtTA binds to P_tet_ to activate gene expression. The important question is how much leakiness was actually prevented by tTS, so that Cre/loxP mediated recombination would only occurs upon Dox treatment.

The major advantage of our two-virus approach is that either single or multiple brain regions can be targeted for inducible and cell type specific gene manipulation by Cre/loxP mediated gene recombination. The major drawback is that a single virus injection can only target a small brain region, but it has the capability to target larger areas by multiple virus injections (Hasan et al., 2013). The inducible genetic switches in our viruses provide an added advantage over a single virus approach for constitutive Cre recombinase expression; with our approach, Cre/loxP mediated gene recombination can be activated by Dox treatment after a particular biological process, such as memory formation, without causing stress to animals by a surgical intervention for virus injection, thus avoiding potential stress-related effects. Given that targeting selective brain region(s) for gene expression/manipulation is a major hurdle with the traditional transgenic, our virus-based approach can be of a great value for neuroscience research and gene therapy.

## Conclusion

In summary, we show here a two-virus approach for inducible gene expression and Cre/loxP mediated gene recombination in the mouse nervous system. Our approach makes it possible to target a single or multiple brain regions, and thus provides the neuroscience community with an important genetic tool to investigate how gene activity, at any particular stage, affects circuit dynamics in various biological functions, including learning and memory processes. It also has great potentials for therapeutic applications.

## Author Contributions

MTH designed experiments, analyzed the data and supervised the project. GKD prepared the viruses, performed the experiments and analyzed the data. MB generated unidirectional constructs and performed experiments. RA performed experiments and investigated the ITR effects. RS provided scientific input, discussion, and resources. GKD and MTH wrote the manuscript.

## Conflict of Interest Statement

The authors declare that the research was conducted in the absence of any commercial or financial relationships that could be construed as a potential conflict of interest.
